# Intersectional equity in health care: assessing complex inequities in primary and secondary care utilization by gender and education in northern Sweden

**DOI:** 10.1186/s12939-020-01272-7

**Published:** 2020-09-11

**Authors:** Fortune N. Nyamande, Paola A. Mosquera, Miguel San Sebastián, Per E. Gustafsson

**Affiliations:** Department of Epidemiology and Global Health, Umeå University, 901 87 Umeå, Sweden

**Keywords:** Intersectionality, Inequality, Inequity, Joint disparity, Referent disparity, Excess intersectional disparity

## Abstract

**Background:**

Knowledge remains scarce about inequities in health care utilization between groups defined, not only by single, but by multiple and intersecting social categories. This study aims to estimate intersectional horizontal inequities in health care utilization by gender and educational level in Northern Sweden, applying a novel methodological approach.

**Methods:**

Data on participants (*N* = 22,997) aged 16–84 years from Northern Sweden came from the 2014 Health on Equal Terms cross sectional survey. Primary (general practitioner) and secondary (specialist doctor) health care utilization and health care needs indicators were self-reported, and sociodemographic information came from registers. Four intersectional categories representing high and low educated men, and high and low educated women, were created, to estimate intersectional (joint, referent, and excess) inequalities, and needs-adjusted horizontal inequities in utilization.

**Results:**

Joint inequalities in primary care were large; 8.20 percentage points difference (95%CI: 6.40–9.99) higher utilization among low-educated women than high-educated men. Only the gender referent inequity remained after needs adjustment, with high- (but not low-) educated women utilizing care more frequently than high-educated men (3.66 percentage points difference (95%CI: 2.67–5.25)). In contrast, inequalities in specialist visits were dominated by referent educational inequalities, (5.69 percentage points difference (95%CI: 2.56–6.19), but with no significant horizontal inequity – by gender, education, or their combination – remaining after needs adjustment.

**Conclusion:**

This study suggests a complex interaction of gender and educational inequities in access to care in Northern Sweden, with horizontal equity observable for secondary but not primary care. The study thereby illustrates the unique knowledge gained from an intersectional perspective to equity in health care.

## Background

The health care system plays a central role in the achievement of equity in health, and implementing laws and policies that aim to foster health equity is therefore seen as a key global objective [1]. Sweden has a reputation of promoting equity in health care, with supporting legislature that places emphasis on needs-based provision of health care [2]. Despite this, there is ample evidence on manifest and growing inequities in the Swedish health care system [3, 4], including in Northern Sweden [5–7], and for different inequity dimensions such as gender [8] and socioeconomic position [7]. However, scarce equity in health research in Sweden and globally has considered complex inequities across multiple intersecting social dimensions. The present study seeks to contribute to this line of inquiry by examining complex or intersectional inequities in health care utilization in Northern Sweden by gender and education, two central and stable dimension of inequity.

Most health care policy and research has given focus to horizontal equity, which is the provision of equal health care to those with equal health care needs regardless of any other factors, such as socioeconomic position, gender, racial/ethnic differences or place of residence [9, 10]. These principles are clearly formulated in Swedish legislation and policies. For example, the Health and Medical Care Act of 1982/2017 highlights that the overarching goal of the Swedish health care system is the provision of good health and care on equal terms for the entire population [11]. The Swedish parliament also agreed in 2003 on a holistic public health policy framework that prioritized health equity, and this emphasis was further strengthened through a revised public health policy bill in April 2018 which specified equitable health as an important goal, and health services as one key target area to reach this goal [12]. The main responsibility for Swedish health care delivery lies at the subnational level of the 21 regions (formerly county councils) [13], and health care is publicly funded through taxes, with comparatively low patient fees and high-cost ceilings. The majority of health care providers remain public, although recent years have seen an increased proportion of private providers particularly in primary health care, as a result of a 2010 reform allowing for establishment of private providers and patients’ freedom to choose their own provider [3]. Primary health care acts as the first health care contact for the population and targets the holistic spectrum of care including health promotion, disease prevention and curative care, and is delivered mainly at local health care centres spread across the country. Specialist care is mostly restricted to hospitals present in the larger towns, and is accessed almost exclusively by referral from the primary health care doctor.

Despite these preconditions promoting equity, multi-faceted health care inequalities have been identified in several Swedish studies. For example, evidence points towards under-utilization of services among those with less financial means, single mothers, foreign-born, and with concerns that health care equity is further threatened by marketization of services [3, 5, 6] [14–18]. At the same time, conventional public health research have been criticized for mainly focusing on analysing the effects of single axes of inequality in health, instead calling for greater application of intersectionality-informed approaches in quantitative public health research, especially research on health inequities [19–28]. Intersectionality theory is premised on how multiple identities and experiences of marginalization interact [29, 30], and highlights how these resultant effects cannot be simply presumed to be equal to the sum of the individual inequalities i.e. non additivity [31]. Intersectionality-informed approaches therefore focus on how multiple simultaneous inequities interact to produce complex inequities in for example population health [19, 20, 32], and intersectional, or complex, inequities thus refers to inequities arising in the combination of multiple inequities, e.g. socioeconomic and gender inequities. The motivation for introducing intersectionality perspectives in equity in health research is to generate evidence that is closer to a complex reality where inequities are highly intertwined. Bauer [25], for example, argued that applying intersectionality provides a more nuanced understanding of the multiple causes, interactions and effects of joint inequalities, and Hankivsky [22] also challenges health researchers to integrate intersectionality in understanding connections between biological and social processes in health care.

Despite this recent interest for intersectionality in research on health equity, most examples of empirical research on intersectional inequities has focused on disparities in health outcomes rather than on health care services or utilization [33–35]. Bastos and colleagues [36] published one of the few studies on intersectionality in health care utilization and how it is patterned by racial disparities. Likewise, whereas multiple novel methodological approaches to analysing intersectional inequalities have been proposed in recent years [37–39], they concern assessment of health inequalities rather than health care inequities. In contrast to the paucity of intersectionality-informed methods for health care inequities, quantitative methods of estimating horizontal inequities have been widely applied in public health research, albeit only in the assessment of “simple” inequities such as income [40] rather than complex inequities. These established methods are based on the principle of adjusting for indicators of health care needs and thereby separating horizontal inequities from inequalities in health care utilization, either by needs-adjustment of conventional regression models or more specialized measures such as the horizontal inequity index [41].

This study seeks to contribute to public health literature by estimating the intersectional inequalities and horizontal inequities in primary and secondary health care utilization by gender and education in Northern Sweden. We will apply a novel methodological approach tailored specifically for this purpose, integrating the method for intersectional inequalities proposed by Jackson [38] with the established approach of needs-adjustment to assess horizontal equity commonly used in health care research on inequities.

## Methods

### Data collection and sampling

The data was collected through the Health on Equal Terms (HET) cross sectional survey of 2014 in four counties/regions in Northern Sweden (Norrbotten, Västerbotten, Västernorrland and Jämtland/Härjedalen). The HET survey has been implemented annually since 2004 by the Public Health Agency in collaboration with Statistics Sweden and the county councils of Sweden. A two-stage probabilistic sampling was undertaken at county and municipal levels to obtain data through postal questionnaires. Individual questionnaire records were linked with register data from Statistic Sweden. The target population was individuals aged between 16 and 84 years inclusive. There were 25,667 responses yielding an overall response rate of 49%. Of these, *N* = 22,997 individuals had valid responses on the main exposure variables (gender and educational level) as well as either of the two outcome variables (GP visits or specialist visits), and were included for analysis. The effective N per variable is reported in Table 1. Ethical approval was obtained from the Regional Ethical Review Board in Umeå.

**Table 1:** Descriptive statistics of key variables by intersectional positions of gender and education

Categories/variable name	Total N (%)	High educated men, N (%)	Low educated men, N (%)	High educated women, N (%)	Low educated women, N (%)
Sample size	22,977 (100%)	5148 (22.40)	5415 (23.60)	6680 (29.10)	5734 (25.00)
*Health care utilization*					
GP visits	21,800				
No	14,728 (67.56)	3555 (71.70)	3412 (67.13)	4364 (67.93)	3397 (63.51)
Yes	7072 (32.44)	1403 (28.30)	1657 (32.69)	2060 (32.07)	1952 (36.59)
Specialist visit	21,592				
No	16,747 (77.56)	3948 (80.10)	3744 (74.40)	5082 (79.94)	3973 (75.33)
Yes	4845 (22.44)	981 (19.90)	1288 (25.60)	1275 (20.06)	1301 (24.67)
*Health care needs*					
Age in years	22,977				
Young age (16–35)	5135 (22.35)	1470 (28.55)	647 (11.95)	2159 (32.32)	859 (14.98)
Middle aged (36–65)	10,687 (46.51)	2420 (47.01)	2514 (46.43)	3531 (52.86)	2222 (38.75)
Old age (66–85)	7155 (31.14)	1258 (24.44)	2254 (41.63)	990 (14.82)	2653 (46.27)
Poor Self-rated Health	22,702				
No	15,464 (68.12)	3891 (76.19)	3310 (61.89)	4918 (74.47)	3345 (59.28)
Yes	7238 (31.88)	1216 (23.81)	2038 (38.11)	1686 (25.53)	2298 (40.72)
Physical Limitations	22,710				
No	13,326 (58.68)	3119 (60.99)	2941 (55.02)	4159 (62.67)	3107 (55.32)
Yes	9384 (41.32)	1995 (39.01)	2404 (44.98)	2477 (37.33)	2508 (44.67)
Chronic Disease	22,977				
No	10,551 (45.92)	2568 (49.88)	2332 (43.07)	3370 (50.45)	2281 (39.78)
Yes	12,426 (54.08)	2580 (50.12)	3083 (56.93)	3310 (49.55)	3453 (60.22)
Poor Mental Health	22,945				
No	17,651 (74.83)	4091 (80.87)	4161 (79.48)	4839 (74.12)	4122 (74.63)
Yes	5294 (22.44)	968 (19.13)	1074 (20.52)	1690 (25.88)	1401 (25.37)

**Fig. 1:**
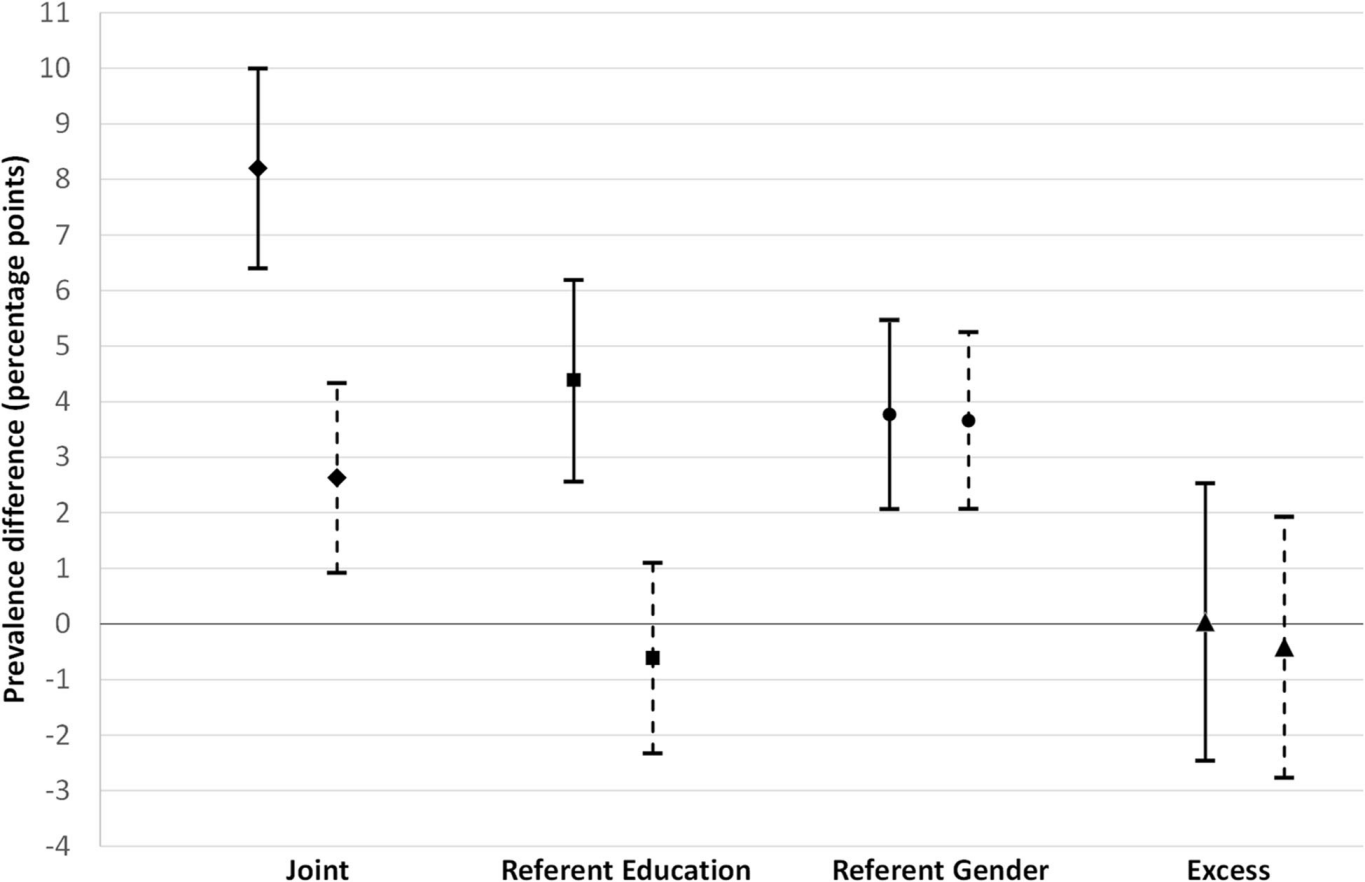
Intersectional inequalities (solid lines) and inequities (dashed lines) in GP utilization in Northern Sweden

### Indicators of health care utilization

To assess health care utilization at the primary health care level, respondents were asked if during the last 3 months they have visited or been visited by a doctor at the health centre. General Practitioner (GP) visits have been used as proxies for primary health care utilization in previous studies on the HET survey [5–7]. Secondary health care utilization was obtained from probing respondents if during the last 3 months they have visited a doctor (specialist) at the hospital. As in previous studies [7], specialist doctor visits have been used as a proxy for the utilization of secondary, hospital-based, outpatient health care services. The responses were coded as (0) for those who reported non-use of general practice/specialist doctors within 3 months prior to the survey, and (1) for those who reported use of these services at any time 3 months prior to the survey.

### Indicators of gender and socioeconomic positions

The variable gender was obtained from registry data and coded as men (0) and women (1). Education was selected as an indicator for socioeconomic position and was also obtained from registry data. It was classified according to the official classification of Statistics Sweden (SUN 2000). The education variable was dichotomized as low (0) (3 years or less of secondary education) and high (1) (more than 3 years of secondary education).

### Intersectional social positions

A combined categorical variable for education and sex was generated by cross-classifying the binary gender and education variables, thus creating four intersectional positions reflecting different combinations of social advantage/disadvantage: *High educated men* representing the doubly socially advantaged group; *low educated women* representing the doubly disadvantaged group; and *high-educated women* and *low-educated men*, both representing middle groups at the intersection of the axis of privilege and of marginalization. Note that distinction of advantage/disadvantage (or privilege/oppression) here is based on the a priori notion of *social* advantage/disadvantage according to the structural dimensions of education and gender, which may or may not correspond to more/less favourable (health care) outcomes.

### Indicators of health care needs

Previous research has highlighted the importance of adjustment for health care needs in attempts to establish horizontal equity in health care [5–7], i.e. to separate disparities in health care utilization due to different needs from inequities in care given equal needs. In this study, health care needs were indicated by age, self-rated health, physical limitation, or illness after accident, chronic diseases and mental health status.

- *Age*: Participants in the age range 16 to 84 were recruited into the study. Age was coded into an ordinal variable young adulthood (1), middle age (2) and old age (3) for the age ranges 16–35, 36–65 and 66–84 respectively.
- *Self-rated health*: The survey participants were asked about how they assessed their state of health. Responses were coded as those who responded good and very good (0) and those who chose fair, poor and very poor (1).
- *Physical limitation or illness after injury:* The responses were coded as those who did not have any physical limitation or discomfort of illness after an injury (0) or those who had a physical limitation or illness after injury (1).
- *Chronic diseases:* The participants were asked if they had any of the following chronic illnesses; asthma, allergy, diabetes and hypertension. Variables for these individual diseases were created and coded as not having the disease (0) and having the disease (1). Those with none of the listed diseases were coded as 0 and those reporting one or more disease were coded as 1.
- *Mental Health*: Self-reported mental health symptoms were obtained using the General Health Questionnaire (GHQ)-12 [42], which has been previously applied in mental health assessments, exhibiting good validity, consistency and detecting depressive disorders [43]. The GHQ-12 has 12 items which cover symptoms during the previous weeks such as lack of concentration, moodiness and sleeplessness, rated on a four-level Likert scale, averaged and multiplied by 12 to create a final index with range of 0–36 [44]. A binary variable was then created with good self-reported mental health coded as good mental health (0) for scores between 0 to 11 and poor mental health (1) for scores greater or equal to 12.

### Statistical analysis

To provide an estimate of the health care utilization disparities we employed as a point of departure the method proposed by Jackson [38], which has been used in recent reports on intersectional inequalities in health-related outcomes [42, 45]. This method examines disparities in a given outcome from a multidimensional perspective, using the most socially privileged group as a point of reference (in this study men with high education). It is based on estimation of four disparities on an additive scale, thus reflecting the absolute gains in the population outcome that would be achieved if the disparity was to be removed [38].

In the present study, the four disparities correspond to the *joint*, the *referent education*, the *referent gender*, and the *excess intersectional* disparities. The joint disparity (JD) is defined as the prevalence difference between the women with low education and men with high education, i.e. the disparity between the doubly underprivileged with the doubly privileged groups. The referent disparity for gender (RD_g_) compares the health care utilization between women with high education and men with high education, and as such assesses the gender disparity among those who are not exposed to education-based disadvantage (e.g. classism). The referent disparity for education (RD_e_) is the difference in health care utilization between men with low education and men with high education, and thus assesses the education-related disparity among those who are not exposed to sexism. Finally, the excess intersectional disparity (EID) is defined as the difference between the JD and the combined sum of the referent disparities (EID = JD – (RD_g_ + RD_e_). The EID is equivalent to an additive scale interaction and therefore provides an absolute estimate of the amount by which the joint disparity surpasses the sum of the two referent disparities.

The above disparities where assessed in two models each for primary health care utilization and secondary health care utilization, respectively. The first model aimed to estimate the crude *inequalities* in utilization. The outcome health care utilization was regressed on the four-category variable for sex and education without adjusting for covariates, and as such reflects the joint, referent and excess intersectional disparities in utilization regardless of the source of this disparity. In theory, unequal utilization could partially or completely be a consequence of differential needs for health care (e.g. pre-existing health inequities) which, while inequitable in and of themselves, would nevertheless signify an equitable utilization of health services as long as utilization is according to needs. The second model therefore specifically aimed to estimate horizontal *inequities* in utilization, i.e. unequal utilization despite equal health care needs. To take into account different health care needs between the four intersectional groups, the second model was therefore additionally adjusted for indicators of health care needs (age, self-rated health, physical limitation after injury or illness, chronic diseases and mental health). Any inequities remaining after adjustment thus reflect the inequitable portion of the crude inequalities in utilization.

Concerning the interpretation of the estimates, a joint or referent disparity greater than zero here reflects greater health care utilization in the respective disadvantaged group compared to the doubly advantaged reference group, while a disparity below zero reflects greater utilization among the doubly advantaged group, without (inequality) or with (horizontal inequity) taking differential health care needs into account. The magnitude of the EID instead reflects the amount by which the disparity between the doubly disadvantaged (women with low education) and doubly advantaged group (men with high education) exceeds what we would expect considering disparities of singly disadvantaged groups together [38]. If the EID is greater than zero, it reflects higher than expected utilization in the doubly disadvantaged group, and if it is below zero, it reflects lower than expected utilization in this group, without (inequality) or with (horizontal inequity) taking potential differential health care needs into account.

Binomial regression was used to estimate the above unadjusted inequalities and needs-adjusted horizontal inequities as prevalence differences (PD) [43], with *p* values and 95% confidence intervals. STATA version 13.1 was used for all the analyses.

## Results

### Descriptive analysis

The characteristics of the study population and the four intersectional groups are summarized in Table 1 below, with 22,997 participants aged between 16 up to 84 years being considered in the statistical analysis. Almost a third (32%) of the respondents had visited a general practitioner at the primary health care centre in the last 3 months. The results further showed that high educated men used primary health care the least (28%) and low educated women utilized primary health care the most (37%), with the middle groups of low-educated men and high-educated women utilizing to a moderate extent (32%). Compared to general practitioners visits and as expected, less people visited specialist doctors at the hospital (22%). However, the utilization of specialist doctors was lowest in both men and women with higher education (20%), and highest among the lower educated groups (approximately 26%).

**Fig. 2:**
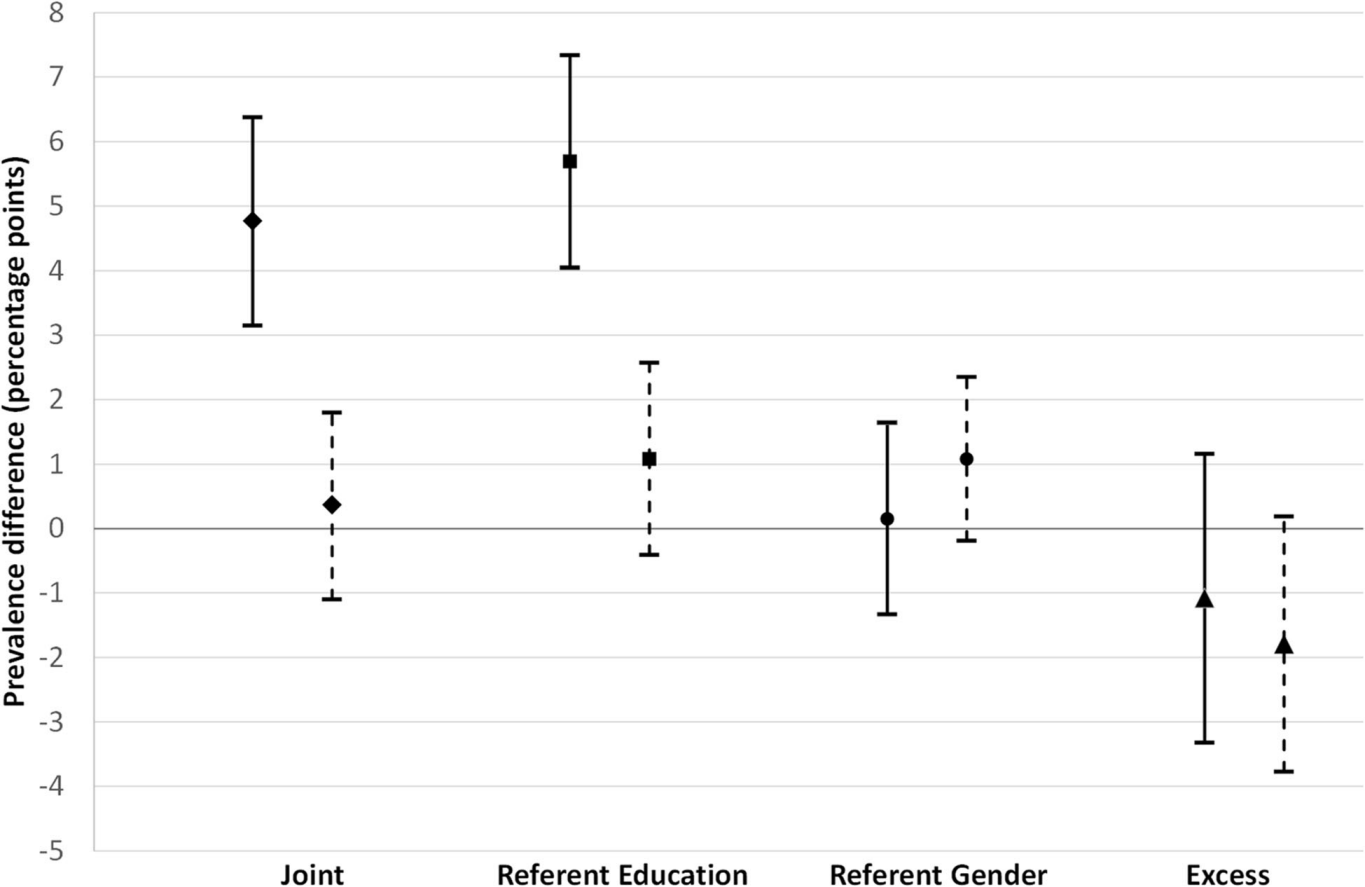
Intersectional inequalities (solid lines) and inequities (dashed lines) in specialist doctor utilization in Northern Sweden

When it comes to the health care needs indicators, diverse patterns were seen across the intersectional groups depending on the indicator. Low-educated women and men displayed worse health profiles for most indicators. High-educated women and men in contrast showed better health, with the notable exception being high-educated women reporting the highest frequency of poor mental health across all intersectional groups.

The results for GP visits as shown in Table 2 and Fig. 1 highlight that the joint disparity in primary health care utilization (i.e. comparing low educated women with high educated men) was 8.2 percentage points (pp), which was reduced to 2.6 pp. (*p* < 0.003) after adjusting for health care needs. The referent gender inequality was 3.8 pp. in the crude analysis and changed minimally to 3.7 pp. (*p* < 0.001) after needs adjustment. The referent education disparity was 4.4 pp. (*p* < 0.001) and was rendered insubstantial (− 0.61 pp) and non-significant (*p* = 0.483) after adjusting for health care needs. Lastly, as shown by the small and non-significant prevalence difference (PD) of excess intersectional inequality (0.034 pp. and *p* = 0.978) and inequity (− 0.42 pp. and *p* = 0.727), the utilization of health care among the doubly disadvantaged groups was reported to the same frequency as we would have expected from considering the educational and gender disadvantages separately.

**Table 2:** Estimating intersectional inequalities and horizontal inequities (needs-adjusted) in GP utilization in Northern Sweden

	Inequalities		Horizontal inequities	
Inequality	Prevalence difference (95%CI)	*P* value	Prevalence difference (95%CI)	*P* value
Joint	8.20 (6.40–9.99)	< 0.001	2.63 (0.92–4.34)	0.003
Referent education	4.39 (2.56–6.19)	< 0.001	−0.61 (− 2.33–1.10)	0.483
Referent gender	3.77 (2.07–5.47)	< 0.001	3.66 (2.07–5.25)	< 0.001
Excess Intersectional	0.034 (−2.46–2.53)	0.978	−0.42 (− 2.77–1.93)	0.727

For specialist visits (as shown in Table 3 and Fig. 2), the joint inequality was in the same direction as for GP visits, although it was markedly smaller (PD = 4.8 pp. as compared to 8.2 pp). However, adjusting for health care needs resulted in a non-significant joint inequity for specialist visits (PD =0.37 pp. and *p* = 0.617). This result thus shows that the differences in specialist doctor utilization between the two intersectional groups defined as high educated men and low educated women were adequately explained by the differential health care needs between them. Unlike for GP visits, the referent gender (comparing high educated women vs high educated men) inequalities (PD =0.15 pp. and *p* = 0.839) and inequities (PD = 1.08 pp. and *p* = 0.097) were small and non-significant before and after health needs adjustment.

**Table 3:** Estimating intersectional inequalities and horizontal inequities (needs-adjusted) in specialist doctors utilization in Northern Sweden

	Inequalities		Horizontal inequities	
Inequality	Prevalence difference (95%CI)	*P* value	Prevalence difference (95%CI)	*P* value
Joint	4.77 (3.15–6.38)	< 0.001	0.37 (− 1.10–1.80)	0.617
Referent education	5.69 (4.05–7.34)	< 0.001	1.08 (−0.41–2.57)	0.156
Referent gender	0.15 (−1.33–1.64)	0.839	1.08 (−0.19–2.35)	0.097
Excess Intersectional	−1.08 (−3.32–1.16)	0.344	− 1.79 (− 3.77–0.19)	0.077

The referent education inequality in specialist visits was 5.7 pp. (slightly higher than for GP visits by about 1 pp) which also became non-significant after adjusting for health care needs (PD = 1.08 pp. and *p* = 0.156). As for GP visits, the excess intersectional disparity was insignificant before (PD = − 1.08 pp. and *p* = 0.344) and after needs adjustment (PD = − 1.79 pp. and *p* = 0.077) showing that the utilization of specialist visits for the low educated women was what we would expect by considering the disadvantages of gender and low education separately.

## Discussion

### Summary of findings

This study estimated complex inequalities and horizontal inequities in primary and secondary health care utilization in Northern Sweden at the intersection of gender and education. The results illustrate the complexity and unique evidence arising from applying an intersectional perspective.

First, we did not find robust evidence for any excess disparity of double (dis)advantage, but rather that the axes of gender and education were independently expressed in health care utilization disparities. Second, low-educated women utilized primary and secondary care considerably more frequently than men, but this inequality was largely (primary care) or completely (secondary care) explained by the greater health care needs of this doubly disadvantaged group. In contrast, high-educated women utilized primary – but not secondary - care to a greater degree than corresponding men, regardless of health care needs. Lastly, the moderately large utilization inequalities rooted in education were completely attributable to different health care needs, both for primary and secondary care. Taken together, the results paint a picture of primary and secondary care in Northern Sweden delivered according to needs when it comes to educational disparities, and with gender inequities disfavouring men remaining in primary care, but which appear equalized at entry to secondary health care.

These findings need to be viewed and interpreted in light of the specific societal and health care context of Sweden, and can be expected to differ in other contexts depending on not only health care organization, financing and provision but also supporting welfare systems such as unemployment and parental leave benefits, the educational system and degree of gender equity in society.

### Inconsistent impact of double disadvantage

One of the original tenets of intersectionality theory relates to the double jeopardy of multiple disadvantage - that “the intersectional experience is greater than the sum” [44]. This notion has remained central in intersectionality-informed quantitative public health [25] and specifically operationalized in manners such as the excess intersectional disparity, originally defined by Jackson [38] and also applied in this study. Whereas we did find notable joint disparities observable throughout the analyses, they were not significantly different from the sum of the two referent disparities of gender and education, thereby not corroborating the double jeopardy hypothesis for these given outcomes and axes of inequality. It should be noted that the double jeopardy hypothesis indeed has been challenged as an oversimplified model, with conflicting empirical support [46] and critique for a simplified focus on “extreme groups” in any given intersectional space [35].

Nevertheless, our results unequivocally demonstrate that the doubly disadvantaged group of low educated women indeed generally report poor health and greater need of health care, which also corresponds to previous intersectionality-informed research on self-reported health from Sweden [32] and other European countries [47]. However, beyond the pre-existing health disparities, we furthermore found that this manifest health disadvantage did not completely explain the high primary care usage in the doubly disadvantaged group (as seen in the joint inequity). For example, despite their quite distinct structural position, health profile, and lower crude utilization of health care, high educated women reported higher primary care utilization even given equal needs (as seen in the referent gender inequity).

### The central role of gender for primary health care utilization

As can be inferred from the discussion above, gender had a profound effect in shaping health care utilization in this study, particularly at the primary care level. This is consistent with other studies that reported higher utilization of health care amongst women as compared to men in high-income context [48–52]. However, it contradicts findings from other intersectionality-informed research from the arguably quite different lower-income context of India, where non-treatment of long-term ailments have been shown to be strongly patterned by gender across all economic classes but to the disadvantage of women instead of men [35].

The share of primary health care utilization not attributable to care needs among low- and high-educated women could possibly be explained by unobserved health care needs specifically relevant to women, such as maternity, gynaecological care and other aspects of women’s health. Previous Swedish studies have indeed found higher primary health care consumption among women than men, even when excluding health care for sex-specific morbidity and reproductive reasons for seeking care [8]. However, the inequalities could also be explained by the impact of lower health care seeking behavior amongst men as compared to women [53], e.g. comparable to the previously reported difficulties to reach and engage Northern Swedish men for health promotion [54] or participation in patient education [55]. In this sense, despite their socially advantaged position, men as a group are disadvantaged from seeking health care due to masculinity norms that may portray them as weak if they seek health care even if they are in need [48], which is also contingent on the intersection with e.g. socioeconomic position [53]. On the other hand, one can also construe this observation as women using health seeking behaviors to successfully leverage the structural disadvantages of gender and low education, and resultantly partly compensate for their poor health.

### Relative equity in specialist care utilization

The absence of horizontal inequities in specialist visits across all the four intersectional categories is in stark contrast to the substantial joint and referent gender inequities in general practitioner utilization. Due to the scarcity of intersectionality-informed studies it is difficult to make direct comparisons to the previous literature, and it is treacherous to make comparisons to studies focusing on only gender or educational inequities in health care utilization. However, one can note that this general inequity pattern contingent on level of care found in our study corresponds to previous Swedish studies reporting a considerably higher primary health care consumption among women than men, but only a marginal gender difference when it comes to specialist outpatient care [8], pro-rich horizontal socioeconomic inequities in primary but not secondary outpatient care [7], and higher utilization of general practitioners among low-income groups but higher utilization of private specialists among high-income groups [56]. The results however contrast to other studies reporting higher educational inequities in outpatient specialist care than in general practitioner visits, e.g. from Norway [57] and across 12 European countries [57].

The comparatively equitable use of specialist visits in our study – both concerning gender, education, and their combination - could be reflective of the underlying forces that determine health care usage at each level of the Swedish health care system. Primary health care utilization is largely contingent on individuals’ own health-seeking behaviour, while access to specialist doctors is almost exclusively based on referrals from the primary health care level. This discrepancy when it comes to responsibility for reaching primary versus secondary care may be further compounded by reforms of primary health care over the last decade which place increased emphasis on the individual patient choices [3]. As such, the access to primary health care is contingent on high- or low-educated women or men’s health literacy and differential health seeking behaviour, as discussed above [54, 55], but when inside the system, both men and women end up accessing specialists more equitably because the decision lies with the primary health care doctors responsible for referrals. In this sense, our results could reflect an ‘equalizing’ effect of referral in the health care system in Northern Sweden that is linking those with greater health care needs at the primary level to specialist care.

On the other hand, it has been shown in multiple reports from Sweden that women are in fact disadvantaged when it comes to various specialist treatments, for example receive less expensive drug prescriptions, older dialysis treatment, later surgery for back pain, less services in case of Alzheimer’s disease, and overall lower quality of care [55]. While these concerns specific health care outcomes not measured in this study, the gender ‘equalization’ apparent in our results at the level of specialist care could in fact reflect that women’s advantage at the primary care level is offset by the challenges faced in specialist care. Here it is also important to note that women’s relative higher health care consumption reflects low-cost care of primary care rather than the high-cost care of specialist care [8].

### Education-related equity in health care utilization

The results showed no education-related intersectional inequities in accessing primary or secondary health care in Northern Sweden. This adds to previous studies on simple, non-intersectional, socioeconomic inequities in health care utilization from the same context, including small horizontal inequities in general practitioner visits, no inequities in specialist visit usage or hospitalizations [7], and among young adults, large income-related but no education-related inequities in youth clinics utilization [5, 6]. A range of studies from other countries, as well as older studies from Sweden [58], have however reported higher health care utilization among high-educated or high-socioeconomic groups; e.g. across 12 European countries [59], and in several Low- or Middle-Income Countries such as Brazil [60], Mongolia [61], Nigeria [62], and Iran [63]. While we indeed found large educational inequalities in both health and health care usage, they were in proportion to each other; i.e. health care utilization was commensurate to need, as posited by the principle of horizontal equity. The Swedish health care system is considered progressive and traditionally framed around the Beveridge model of health care financing, where health care is financed by general taxation thus promoting universal health access. Even though there has been a successively increased market-orientation and privatization of Swedish primary health care that may impact negatively on health care equity [3], Northern Sweden has been a region less affected by these developments [64]. We conclude that health care at the primary health care level was utilized according to needs amongst intersectional groups of different educational level in this study. This finding could reflect the inherent impact of universal health coverage mitigating classism in the health system.

### Methodological considerations

Although this study proposes a refinement to existing quantitative methods in assessing intersectionality in health care, we have noted some limitations that should be considered.

First, the response rate was 49%, which is comparable to most studies conducted in the same setting with reliable results. The demographic and social characteristics of the non-respondents (e.g. age, gender, education, area of residence) were not available and the extent of any selection bias could therefore not be assessed. Selection bias is therefore an uncontrolled threat against internal validity, and which could lead to either over- or underestimation of the studied inequities. Moreover, we cannot draw any causal inferences from our study as our data was collected from a cross-sectional survey.

While the proposed analytical method produce an appropriate assessment of intersectional inequities on a familiar additive scale, which are not captured by conventional methods for horizontal inequities along single dimensions [41], it has limitations. Most importantly, it is developed and has only been used for two binary inequity dimensions [38], which limits the scope of the analysis. While it technically could be extended to include more inequity dimensions (e.g. ethnicity, sexual orientation, geography, and disability), conceptualization, estimation and interpretation of the individual inequities becomes increasingly challenging. If the aim is to estimate a large set of inequity dimensions, other intersectionality-informed methods developed for this purpose might be more suitable [37]. Moreover, estimation is more straightforward for continuous compared to binary outcomes [38], a limitation that however is even more pronounced for alternative methods [37]. The method is furthermore based on adjustment for health care needs to assess horizontal inequity, and consequently, there is a risk of underestimation of health care needs as it is theoretically impossible to capture all health care needs. For instance, and as noted above, we could not provide adjustments for women’s health needs such as maternal health care needs, gynaecological requirements or other women reproductive health care needs, as this information was not available in the survey data. Nevertheless, we tried to capture several facets of health care needs that have also been applied in previous literature [5–7].

## Conclusions

The present study employed an intersectional approach to assess horizontal inequity by gender and educational level in Swedish primary and secondary outpatient care. The study suggests that whereas utilization of specialist care in Northern Sweden roughly follows the principle of horizontal equity along and across these two axes of inequality, men seem to be disadvantaged when it comes to primary care utilization given their health care needs. This suggests that the mode of access to specialist care in the Swedish health care system may work in an equalizing manner, largely compensating for initial inequities when accessing primary care. The study also illustrate how structurally advantaged groups may be entangled in complex processes that may not be captured by traditional assessment of inequalities or horizontal equity. Swedish health care policy makers and researchers therefore need to pay attention to intersectional inequities that can be perceived to be advantaged, and targeting pathways to accessing primary care, for example health promotion messages that addresses masculinity norms of poor perceptions of health risk, severity of illness and low need for health care among men. Moreover, greater attention needs to paid to instruments adequately capturing health care needs of women when estimating of horizontal equity among intersectional groups.

## Data Availability

Access to the data used in the current study is managed by the register holders, the respective County Council/Region, and data are as such not publicly available.

## Abbreviations

HET: Health on Equal Terms
GP: General Practitioner
GHQ: General Health Questionnaire
JD: Joint disparity
RDg: Referent disparity for gender
RDe: Referent disparity for education
EID: Excess intersectional disparity
PD: Prevalence difference
